# Applying Amide Proton Transfer-Weighted Imaging (APTWI) to Distinguish Papillary Thyroid Carcinomas and Predominantly Solid Adenomatous Nodules: Comparison With Diffusion-Weighted Imaging

**DOI:** 10.3389/fonc.2020.00918

**Published:** 2020-06-19

**Authors:** Guomin Li, Guihua Jiang, Yingjie Mei, Peng Gao, Ruijian Liu, Min Jiang, Yue Zhao, Meng Li, Yunfan Wu, Shishun Fu, Mengchen Liu, Liming Li, Wuming Li, Jianhao Yan

**Affiliations:** ^1^The Second School of Clinical Medicine, Southern Medical University, Guangzhou, China; ^2^The Department of Medical Imaging, Guangdong Second Provincial General Hospital, Guangzhou, China; ^3^Philips Healthcare, Hong Kong, China; ^4^Department of General Surgery, Guangdong Second Provincial General Hospital, Guangzhou, China

**Keywords:** papillary thyroid carcinoma, predominantly solid thyroid adenomatous nodule, amide proton transfer (APT), diffusion-weighted imaging (DWI), differentiation

## Abstract

**Background:** Amide proton transfer-weighted (ATPw) imaging is a novel MRI technique that has been used to identify benign and malignant tumors. The present study evaluated the role of APTw imaging in differentiating papillary thyroid carcinoma from predominantly solid adenomatous nodule.

**Methods:** This study included 24 cases of solitary papillary thyroid carcinoma, and 20 cases of solid adenomatous nodules. Normal thyroid tissues were examined in 12 healthy subjects. The healthy subjects, eight cases of adenomatous nodule with cystic degeneration, and 12 cases of thyroid goiter, were only considered in the descriptive analysis, not included in our statistical analysis. The mean APTw value and the apparent diffusion coefficients (ADCs) of papillary thyroid carcinoma and solid adenomatous nodule were compared via a Mann-Whitney U test and receiver operating characteristic (ROC)-curve analyses.

**Results:** The adenomatous nodule (3.3 ± 1.3%) exhibited significantly higher APTw value (*p* < 0.05) than that of the papillary thyroid carcinoma (1.8 ± 0.7%). The optimal cut-off value of the mean APTw value in differentiating papillary thyroid carcinoma from adenomatous nodule was 3.15%, with a sensitivity of 60% and a specificity of 100%. The mean ADC of papillary thyroid carcinoma (1.2 ± 0.2 × 10^−3^ mm^2^/*s*) was significantly lower than that of adenomatous nodule (2.0 ± 0.4 × 10^−3^ mm^2^/s). The optimal cut-off value of the mean ADC was 1.35 × 10^−3^ mm^2^/s, with a sensitivity of 100% and a specificity of 75%. Based on the ROC-curve analysis of APT and ADC, the ADC showed a higher area under the curve (AUC) than that of APT (AUC_APT_ = 0.84, AUC_ADC_ = 0.95).

**Conclusion:** APTw imaging may be as useful as DWI for the differentiation of papillary thyroid carcinoma from predominantly solid adenomatous nodule. Although the sensitivity of ADC was greater than that of APT, APT had greater specificity.

## Introduction

Thyroid nodules are becoming increasingly prevalent. Nodular goiters and adenomas are the most common benign thyroid nodules, and papillary thyroid carcinoma is the most common malignant thyroid tumors (1). Nodular goiters and adenomas are usually treated by clinical observation, especially in the elderly. In contrast, the optimal treatment for papillary thyroid carcinoma is surgical excision. Therefore, the precise preoperative differentiation of nodular goiter or adenoma and papillary thyroid carcinoma is of significant practical relevance. Adenomas can occur alone or in combination with nodular goiters. Their morphologies, signals, and enhancements are similar, often resulting in difficult differential diagnoses. In particular, solitary solid nodular goiters are challenging to identify with adenomas. As such, we used adenomatous nodules (2–4) to replace solitary solid nodular goiters or thyroid adenomas in the present study.

Amide proton transfer-weighted (ATPw) imaging is a novel magnetic resonance imaging (MRI) technique that can detect mobile proteins and peptides that contain abundant amide (-CO-NH-) chemical constituents (5, 6). The APTw values can reflect the concentrations of mobile macromolecules, such as proteins and peptides. Early reports of APTw imaging for cancer assessment have focused on the brain. According to the previous literature, high-grade gliomas show higher APTw values than low-grade gliomas (7, 8), and APTw imaging is useful for assessing tumor aggressiveness. Investigators in recent human studies have reported preliminary APT findings in the breast (9), prostate (10), cervix (11), rectum (12), and lung (13). APTw values were higher in cancers than in normal tissues or benign tumors, and APT levels varied between different malignant tumors groups or different histological grades. Furthermore, APT may provide additional information to improve the results of diffusion-weighted imaging (DWI) or other MRI techniques.

The head-neck regions are challenging for molecular MRI techniques because of magnetic field inhomogeneity, and motion and such tissues are prone to artifacts. In a preliminary study on the characterization of head and neck tumors which showed the feasibility of performing APTw imaging in the head and neck by using a technique adapted from the brain, the authors hypothesized that malignant tumors have higher APT levels than healthy tissues and benign tumors and that APT levels differ among malignant tumor groups. They studied the patients with nasopharyngeal undifferentiated carcinoma, squamous cell carcinoma, non-Hodgkin's lymphoma, and benign salivary gland tumors (14, 15).

We previously reported on a study about patients with thyroid tumors that showed the feasibility of performing APTw imaging in the neck. The results showed that the APTw values of malignant nodules of the thyroid are lower than that of benign nodules, which is different from other tumors (16). However, thyroid tumors are prone to cystic change (17), which have a significant influence on the measurement of APTw values. Our previous study samples were simply divided into benign groups and malignant groups. Both the two groups contained different pathological types, and cystic nodules were not excluded. We want to explore the diagnostic performance of APTw imaging in differentiating papillary thyroid carcinoma from predominantly solid adenomatous nodule. Now we need to further group and measure them accurately, calculate the threshold, sensitivity, and specificity of APT and ADC to distinguish solid papillary thyroid carcinoma and solid adenomatous nodule.

## Materials and Methods

### Subjects

The local Institutional Review Board approved this study, and all subjects gave written, informed consent before participation in this study. Between 2018 and 2019, 24 biopsy-proven papillary thyroid carcinomas, 28 cases of adenomatous nodule, and 12 cases of thyroid goiter underwent MRI exam. This study included 12 healthy subjects. The healthy subjects, 8 cases of adenomatous nodules with cystic degeneration, and 12 cases of thyroid goiter were only considered in the descriptive analysis, not included in statistical analysis. Thus, 24 papillary carcinomas (15 females, 9 males; 41.16 ± 13.43 years old; range, 29–68 years old) and 20 adenomatous nodules (13 females, 7 males; 42.80 ± 10.20 years old; range, 22–72 years old) were included in the study.

### MRI Protocols

MR imaging was performed with a Philips 3-Tesla (3T) scanner (Ingenia, 3.0 T; Philips Medical Systems, The Netherlands). A 16-channel head-neck coil was used for scanning. The patients underwent T1- [repetition time (TR)/echo time (TE), 570/18 ms] and T2-weighted MR imaging [TR (ms)/TE (ms), 2,500/100] with the section thickness of 4 mm, an intersection gap of 1 mm, field-of-views of 20–25 cm, and an acquisition matrix of 256 × 224. The scan time of T1WI is 85 s and the scan time of T2WI is 150 s. Images were obtained in axial and coronal planes, following scout images in the sagittal plane.

In addition to conventional MR imaging (T1-weighted imaging, T2-weighted imaging, and Gd-enhanced T1-weighted imaging), APTw sequences and reduced field-of-view (r-FOV) diffusion-weighted sequences with different b values (0, 800 mm^2^/s) were acquired. Other parameters of DWI were as follows: field-of-views of 116 × 51 mm^2^; voxel size of 1.81 × 1.81 mm^2^; slice thickness of 4 mm; TR (ms)/TE (ms) of 3,687/62; scan time of 221 s. APTw imaging was performed using a 3-dimensional (3D) turbo-spin-echo Dixon sequence with these parameters as follows: slice thickness of 4.4 mm, acquisition voxel size of 1.8 × 1.8 mm^2^, TR (ms)/TE (ms) of 4,108/5.9, scan time of 259 s, and turbo spin-echo factor of 158. APTw imaging was performed with seven saturation-frequency offsets (offsets = ± 2.7, ± 3.5, ± 4.3 ppm, and 1,540 ppm). The protocol was repeated three times at ±3.5 ppm to increase the signal-to-noise ratio within an appropriate time frame. Saturation radio-frequency pulses for APTw imaging were implemented with an amplitude of 2 μT and a duration of 2 s. B_0_ maps were obtained with three acquisitions at 3.5 ppm of different echo times. B0-corrected ATPw images were reconstructed online.

### Imaging Analysis of APT and Apparent Diffusion Coefficients (ADCs)

The two radiologists conducting the present study determined by consensus whether the APTw maps and ADC maps were acceptable for statistical analysis. All images were interpreted by two radiologists specializing in head and neck imaging. APTw and ADC imaging were automatically generated via a Philips post-processing workstation. We calculated the mean APTw value and ADC value by drawing a region of interest (ROI). The radiologists drew an ROI around the predominantly solid thyroid nodules or drew a ROI on the central of one leaf of the normal thyroid tissues on the APTw image and ADC map by using the T2WI for reference, and then the mean APTw value and mean ADC value was obtained from the ROI, as shown in **Table 2**. The ROI analysis was repeated by two observers to assess the inter-observer agreement. The two radiologists processed the MR images independently. They were blinded to the histopathologic data.

### Statistical Analysis

The APTw values and ADC values of the papillary thyroid carcinoma were compared with that of the thyroid adenomatous nodules using a Mann-Whitney U test. The diagnostic performances of significant APTw parameters for differentiating the papillary thyroid carcinoma from the adenomatous nodules were assessed by using ROC-curve analyses with the AUC. The APTw threshold was acquired by calculating the Youden index, which is the sum of the sensitivity and specificity −1, and the APTw value corresponding to the point where the Yoden index is the largest was considered the APTw threshold. Then the sensitivity, specificity of the optimal thresholds were calculated. Statistical analysis was performed using SPSS software 21.0. All statistical tests were two-sided, and a *p*-value of <0.05 was considered to indicate a statistically significant difference.

## Results

**Figure 1:**
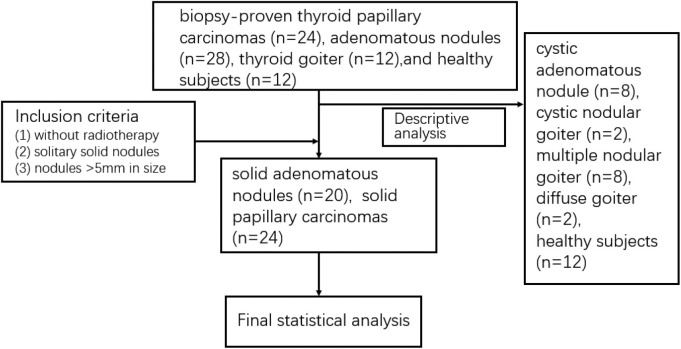
Subject selection flowchart.

**Figure 2:**
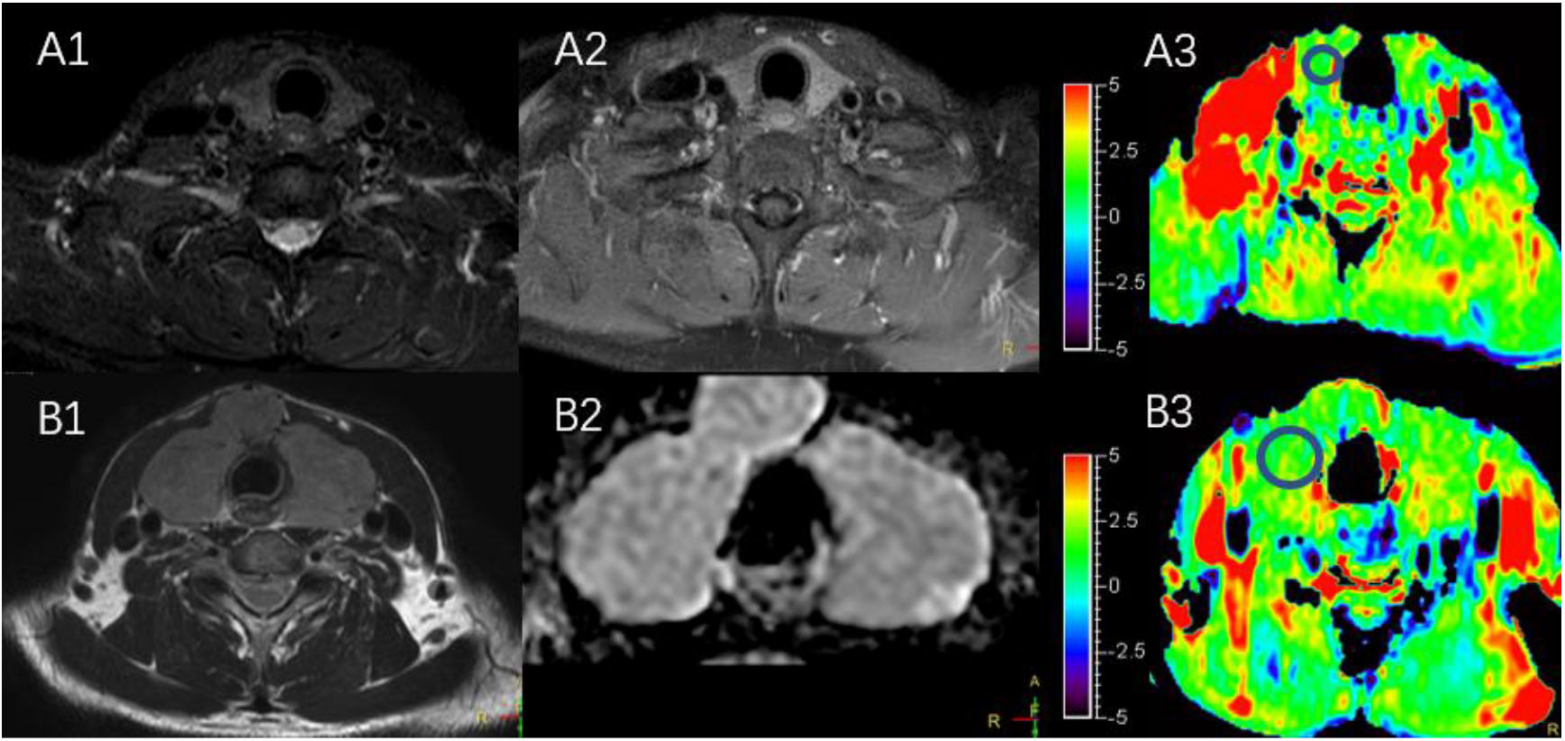
**(A1–A3)** MR images of a normal thyroid, including T2WI **(A1)**, Gd-T1WI **(A2)**, and APTWI **(A3)**. **(B1–B3)** MR images of diffuse goiter, including T2WI **(B1)**, ADC map **(B2)**, and APTWI **(B3)**.

**Figure 3:**
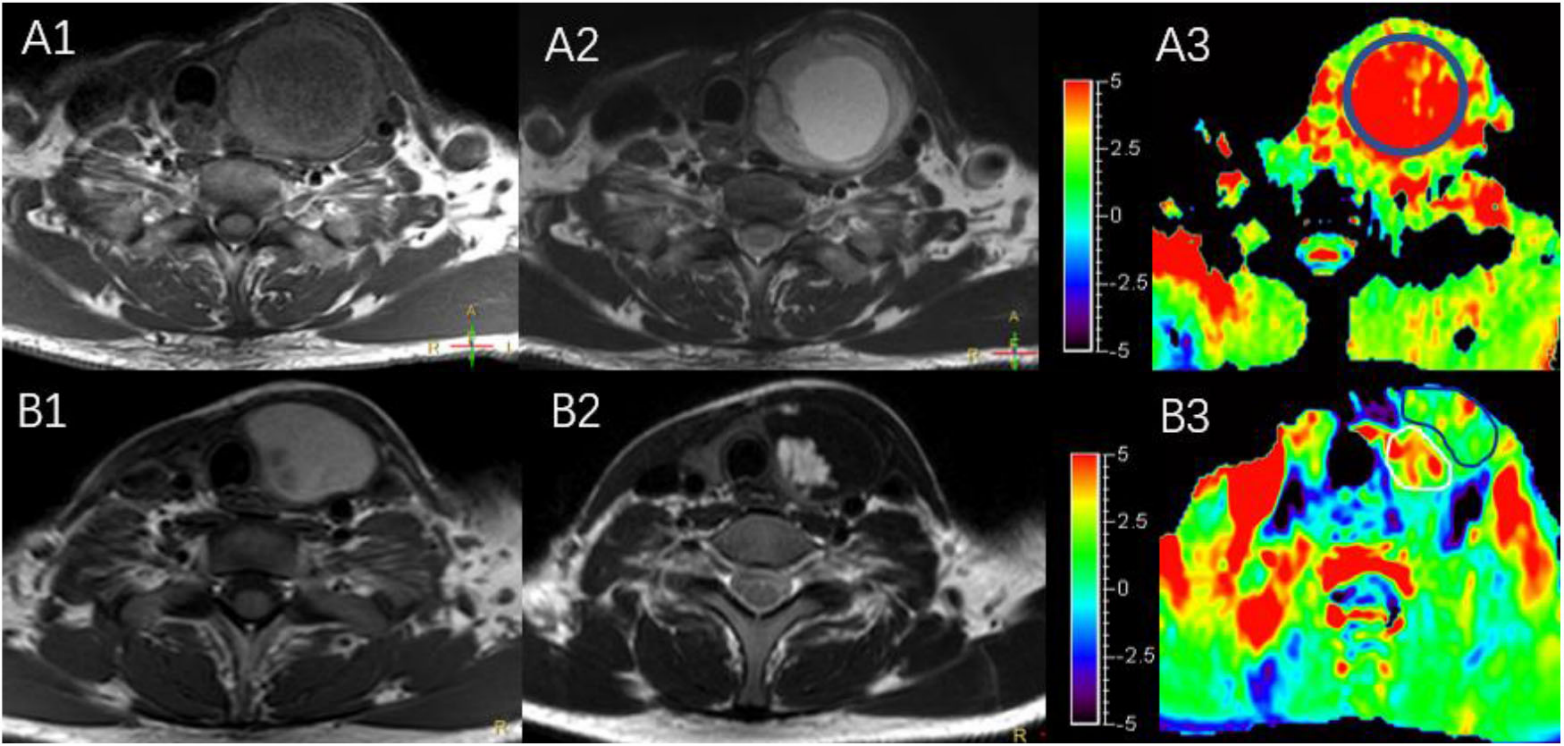
MR images of two predominantly cystic thyroid nodules, including T1WI **(A1,B1)**, T2WI **(A2,B2)**, and APTWI **(A3,B3)**.

The characteristics of the patients are shown in Table 1 and the subjects selection flowchart is shown in Figure 1. We first assessed the radiographic features of some interesting cases and normal thyroids using several standards sequences (T1-weight images, T2-weight images, Gd- T1-weight images, DWI) and APTw sequences. Figure 2 shows the normal thyroid tissue and diffuse goiter. They appear homogenously isointense on APTWI, and their APTw values (normal thyroid, 2.15%; diffuse goiter, 2.36%) are similar, and neither is very high. Figure 3 shows two thyroid nodules with cystic changes. The A cyst rich in serous fluid and appeared hypointense on T1-weight images (T1WI), hyperintense on T2-weight images (T2WI), and hyperintense on APTWI (APTw values = 7.33%). The B cyst is rich in thyroid colloid and appears hyperintense on T1WI, hypointense on T2WI, hypointense on APTWI (APTw values = 1.53%). The solid portion appears isointense on T1WI and hyperintense on T2WI and APTWI (APTw values = 3.56%).

**Table 1:** Patient characteristics and pathologies.

**Pathology**	**No**.	**Female:male**	**Age (years)**
Adenomatous nodule	20	13:7	43 ± 10
Papillary carcinoma	24	15:9	41 ± 13
Total	44	28:16	42 ± 12

**Figure 4:**
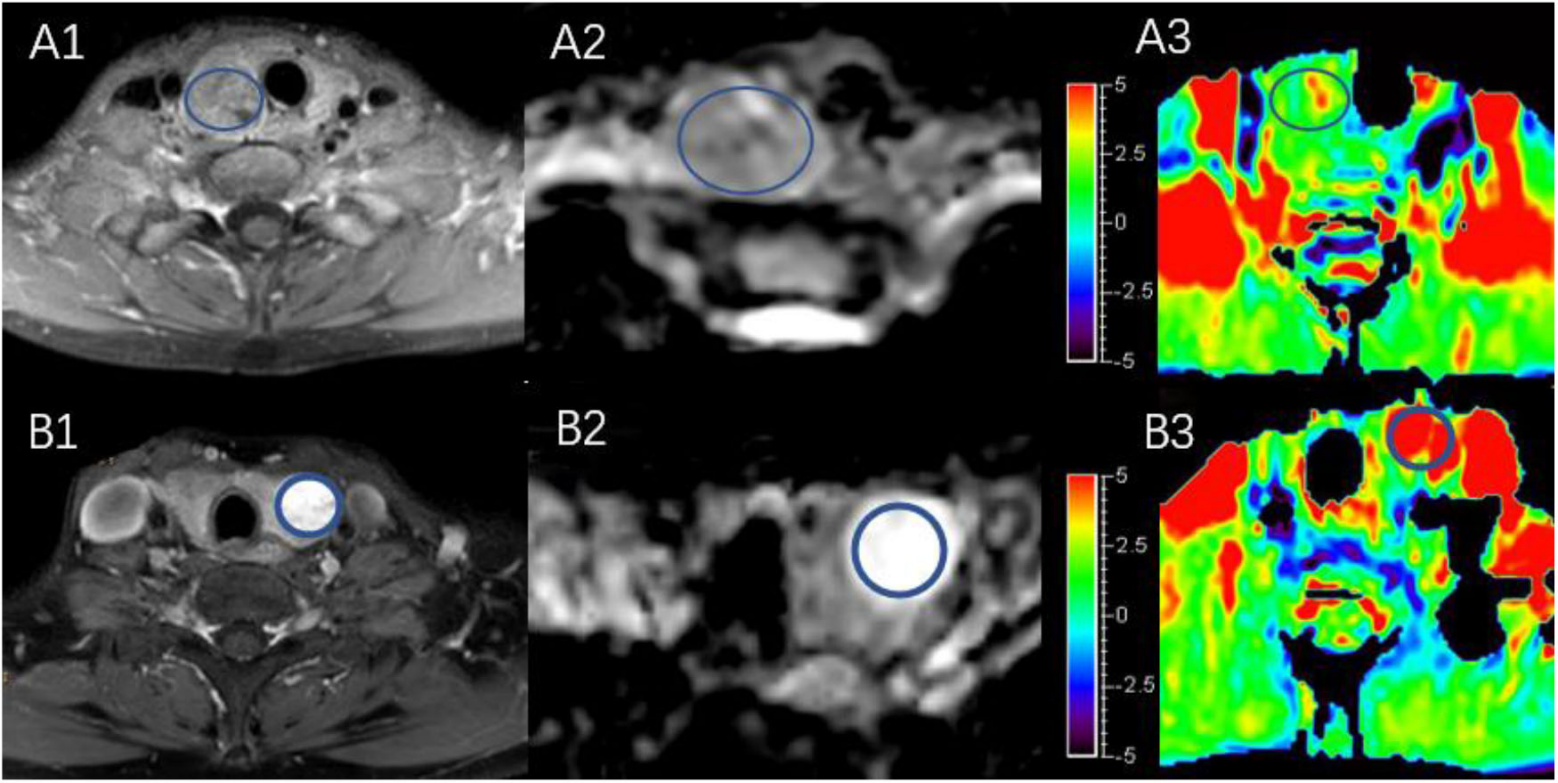
Both **(A,B)** are predominantly solid adenomatous nodules on Gd-T1WI **(A1,B1)**, ADC map **(A2,B2)**, and APTWI **(A3,B3)**.

Figure 4 shows two predominantly solid adenomatous nodules. One is an atypical adenomatous nodule and appeared mild enhancement on Gd-T1WI, mild hyperintense on an ADC map, and isointense on APTWI (APTw values = 2.05%). The other is a typical adenomatous nodule and exhibited strong enhancement on Gd-T1WI and hyperintense on the ADC map and APTWI (APTw values = 5.21%).

**Figure 5:**
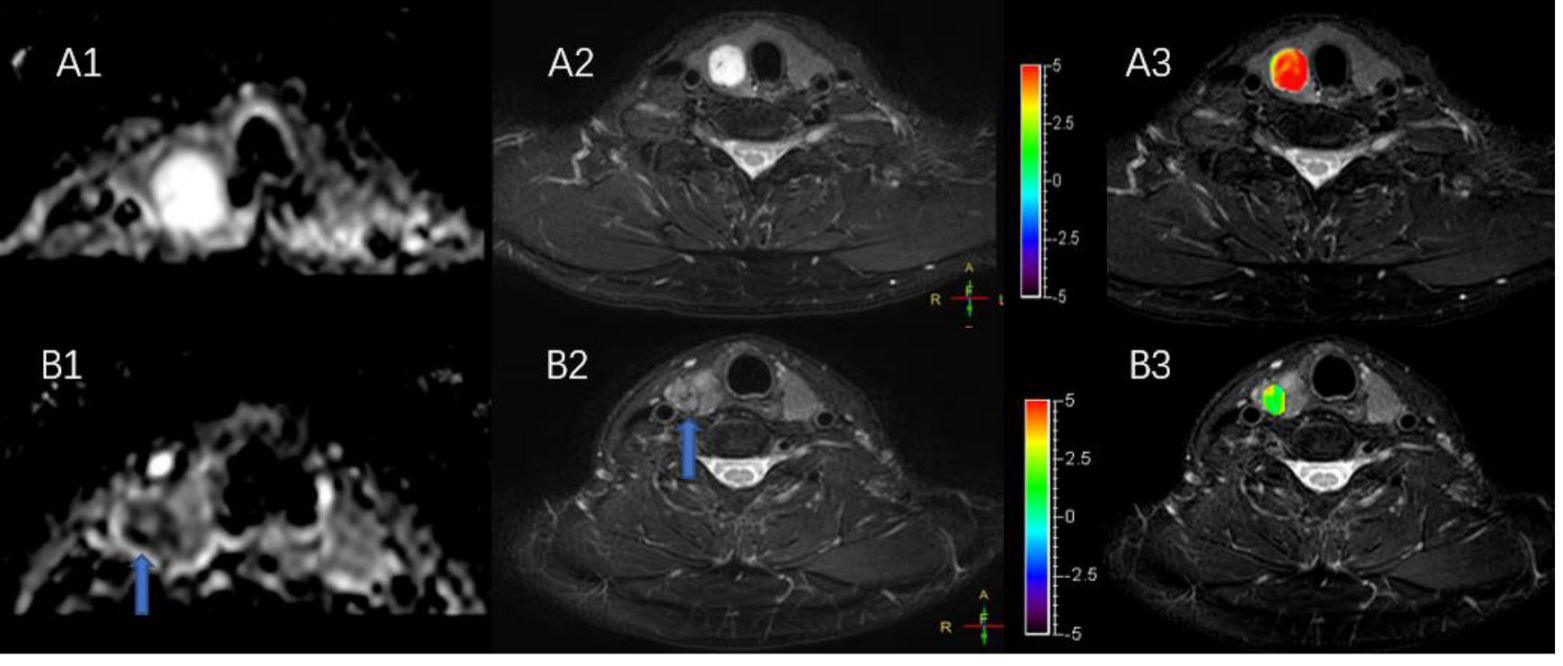
**(A,B)** Show a solid adenomatous nodule and a papillary thyroid carcinoma on the ADC map **(A1,B1)**, T2WI **(A2,B2)**, and the combination of T2WI and APTWI **(A3,B3)**.

Figure 5 shows a typical solid adenomatous nodule and a papillary thyroid carcinoma. The adenomatous nodule appeared hyperintense on both the ADC map and T2WI, and the mean APTw value was 6.10%. The papillary thyroid carcinoma appeared hypointense on ADC map, heterogeneous iso-/hypo-intensity on APTWI, and the mean APTw value was 1.93%.

**Figure 6:**
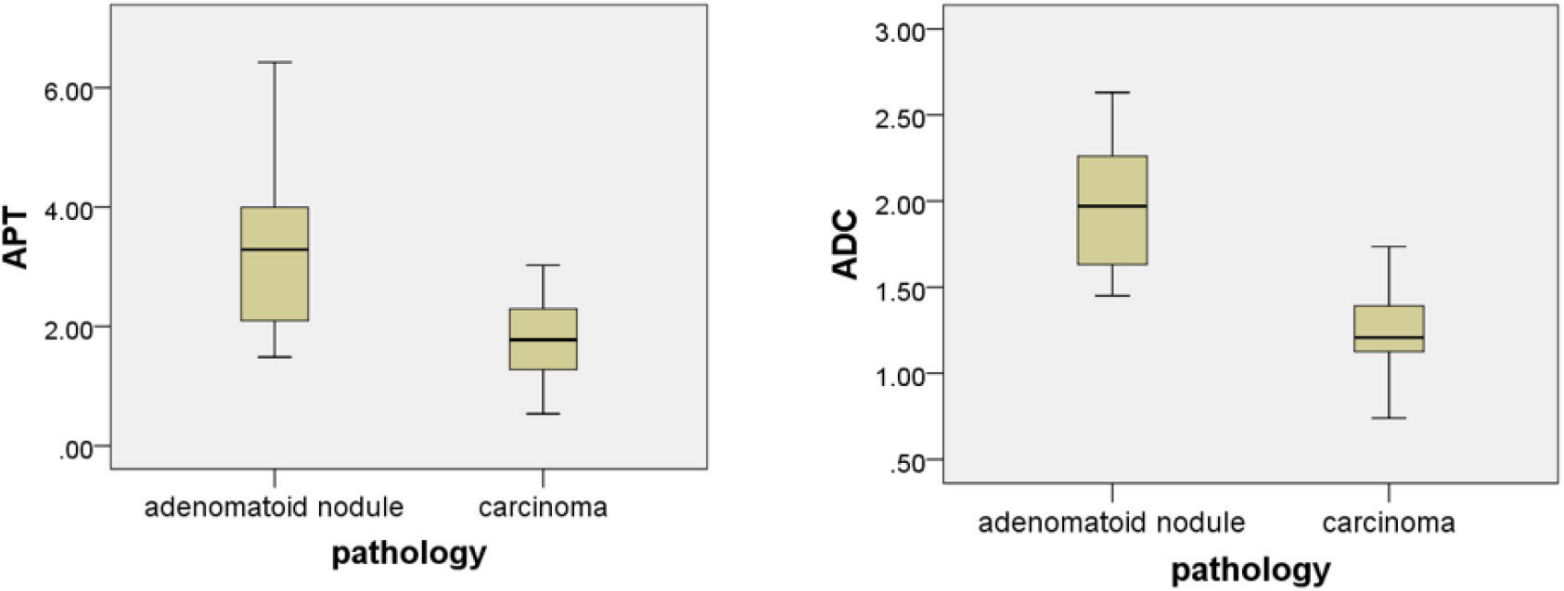
Box plot of the APT (%) and the ADC (mm^2^/s) of adenomatous nodule and papillary thyroid carcinoma.

**Figure 7:**
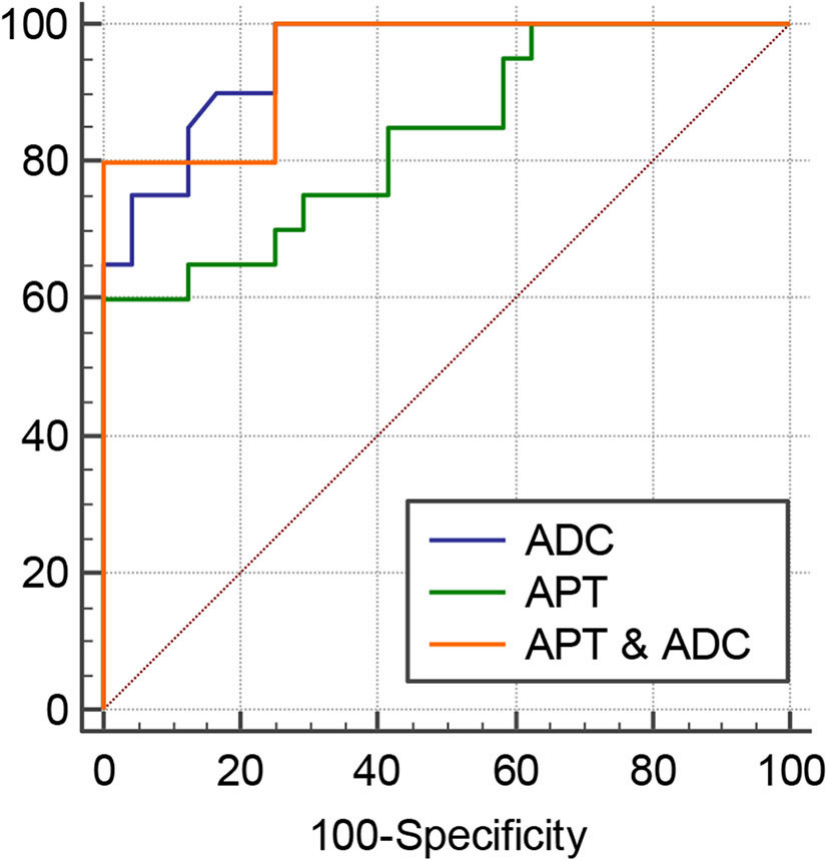
ROC curves of APT, ADC, and the combination of APT and ADC for differentiation between papillary thyroid carcinoma and adenomatous nodule: the AUC was 0.95 (ADC), 0.95 (APT and ADC), and 0.835 (APT).

The intraclass correlation coefficients (ICC) showed excellent observer agreement (ICC_APT_ = 0.92, ICC_ADC_ = 0.96, *p* < 0.01). Table 2 and Figure 6 show the APTw values and ADC values of thyroid nodules in this study, and there was a significant difference in the APTw value and ADC of the papillary thyroid carcinoma and adenomatous nodule. The adenomatous nodule (3.3 ± 1.3%) exhibited higher APT-weighted signal intensities than that of papillary carcinoma (1.8 ± 0.7%; *p* < 0.01). The mean ADC of the papillary thyroid carcinoma (1.2 ± 0.2 × 10^−3^ mm^2^/s) was significantly lower than that of the adenomatous nodule (2.0 ± 0.4 × 10^−3^ mm^2^/s; *p* < 0.01). The optimal cut-off value of the mean APTw value in differentiating papillary thyroid carcinoma from the adenomatous nodule was 3.15%, with a sensitivity of 60% and a specificity of 100% (Figure 7). The mean ADC of the papillary thyroid carcinoma was significantly lower than that of the adenomatous nodule. The optimal cut-off value of the mean ADC in differentiating papillary carcinoma from adenomatous nodule was 1.35 × 10^−3^ mm^2^/s, with a sensitivity of 100% and specificity of 75%. The ROC curve analysis revealed that ADC exhibited a higher AUC value compared to that of APT (AUC_APT_ = 0.84, AUC_ADC_ = 0.95). The r-FOV DWI showed a better diagnostic performance than that of APTw imaging. Although the sensitivity of DWI (100%) was significantly higher than that of APT (60%), the specificity of APT (100%) was substantially higher than that of ADC (75%).

**Table 2:** The APTw values and ADC values of thyroid nodules.

**Pathology**	**APTw value (%)**	**ADC (mm^**2**^/s)**	**Diameter (mm)**	***p*-value**
Adenomatous nodule	3.3 ± 1.3	2.0 ± 0.4	24 ± 9	<0.001
Papillary carcinoma	1.8 ± 0.7	1.2 ± 0.2	11 ± 5	

## Discussion

In this study, we explored the diagnostic performance of using APTw imaging to differentiate papillary thyroid carcinoma from the solid adenomatous nodule. The aim was to differentiate papillary thyroid carcinoma from adenomatous nodule so that the patients with papillary thyroid carcinoma would be able to receive appropriate treatment at an earlier stage while avoiding unnecessary surgery in the patients with adenomatous nodules. The present study showed a significant difference between the ADC and APTw value of papillary thyroid carcinoma and adenomatous nodule, in which the most adenomatous nodules had higher mean ADC and APTw value than papillary thyroid carcinoma. It is not clear why adenomatous nodules have higher APTw value than papillary thyroid carcinoma, as opposed to other tumors.

Diffusion-weighted imaging provides a better characterization of tissues because it can reflect the random motion of water molecules, which is disturbed by intracellular macromolecules. Previous studies have evaluated the role of diffusion-weighted imaging in differentiating benign from malignant thyroid nodules (18–24). The APTw values can reflect the concentrations of mobile macromolecules, such as proteins and peptides. Our previous studies on the thyroid established a positive correlation between APTw values with ADC. Part of the reason for this may be because the APTWI detects free protein rather than solid proteins.

As shows in Figure 2, the APTw value of normal thyroid tissue and diffuse goiters are similar, and neither is very high despite relatively abundant colloid components in the diffuse goiter. The components of the cystic thyroid zone consist mainly of serous fluid, thyroid colloid (thyroglobulin), and blood from different periods, and they exhibit characteristic MR signals (25, 26). Serous fluid often appears hypointense on T1WI and hyperintense on T2WI, similar to that of water. Thyroid colloid contains macromolecular thyroglobulin, which shortens the T1 relaxation time and shows a homogenous high signal on T1WI. Blood fluid from different periods can display various heterogeneous signal intensities (27, 28). Figure 3 exhibits a thyroid nodule with the cystic change, and the components of the cystic thyroid zone consist mainly of thyroid colloid, but the APTw value is low. It is speculated that the thyroid colloid does not show a high signal intensity on APTWI, and the reasons why the APTw value of adenomatous nodule was higher than that of papillary thyroid carcinoma is not that adenomatous nodule contains abundant thyroid colloid.

In the present study, most solid adenomatous nodules showed significantly high APTw value, but some were similar to normal thyroid tissue. The typical adenomatous nodule that showed high signal on the APTw image exhibited isointense on T1WI, hyperintensity on T2WI, and strong enhancement on Gd-T1WI, indicating that there is abundant microvessel on the typical adenomatous nodule. The typical adenomatous nodule had a high ADC value, indicating active water-molecule movement. On the contrary, the atypical adenomatous nodule exhibited isointense on the ADC map and slight enhancement on Gd-T1WI, indicating the restricted water-molecule movement and the less microvessel compared with typical adenomatous nodule (29). The blood supply of papillary thyroid carcinoma is not as abundant as in typical adenomatous nodule, and papillary thyroid carcinoma has a high density of tumor cells, small extracellular space, and high cytoplasmic viscosity (21, 30–34). In conclusion, abundant blood supply may underlie why adenomatous nodule has higher APTw value than papillary thyroid carcinoma.

The present study had some limitations. First, the sample size was small. Second, the head and neck are challenging regions in which to perform functional MRIs because of field inhomogeneity, relatively low signal-to-noise ratio, movement artifacts, and difficulties with imaging fat suppression. Third, some thyroid microcarcinomas may occur in adenomatous nodules. In addition, there is some biases because the ROIs were drawn manually on the APTWI and ADC maps by using the anatomic images for reference. The APT value of adenomatous nodules is not absolutely higher than that of papillary thyroid carcinoma, but it is because most papillary carcinomas are relatively small when they are found. At this stage, the papillary carcinoma has incompletely developed blood vessels and relatively less blood vessels. If the supply of blood vessels to the papillary cancer in the late stage becomes rich, then like other malignant tumors, the APT value of papillary thyroid carcinoma will increase and close to the adenomatous nodule.

## Conclusions

APTw imaging may be useful for the differentiation of papillary thyroid carcinoma from predominantly solid adenomatous nodule. DWI had higher accuracy and sensitivity but lower specificity than APTw imaging. From our present results, we hypothesize that plentiful blood supply may be the main reason why the APTw value of the typical adenomatous nodule is higher than that of papillary thyroid carcinoma.

## Data Availability Statement

The datasets generated for this study are available on request to the corresponding author.

## Ethics Statement

The studies involving human participants were reviewed and approved by Ethics committee of Guangdong Second Provincial General Hospital. The patients/participants provided their written informed consent to participate in this study.

## Author Contributions

GJ designed the experiment. GL and RL carried out the experiment. LL, WL, PG, YZ, MJ, and MLi collected and sorted out the data. YW, SF, and MLiu helped on data management and processing. JY and GL wrote the manuscript. YM revised the manuscript.

## Conflict of Interest

YM was employed by the company Philips Healthcare. The remaining authors declare that the research was conducted in the absence of any commercial or financial relationships that could be construed as a potential conflict of interest.
